# Biomarker discovery in attention deficit hyperactivity disorder: RNA sequencing of whole blood in discordant twin and case-controlled cohorts

**DOI:** 10.1186/s12920-020-00808-8

**Published:** 2020-10-28

**Authors:** Timothy A. McCaffrey, Georges St. Laurent, Dmitry Shtokalo, Denis Antonets, Yuri Vyatkin, Daniel Jones, Eleanor Battison, Joel T. Nigg

**Affiliations:** 1 Division of Genomic Medicine, Department of Medicine, The George Washington University, 2300 Eye St., Washington, DC 20037 USA; 2 The St. Laurent Institute, Vancouver, WA USA; 3 A.P. Ershov Institute of Informatics Systems, Novosibirsk, Russia; 4 AcademGene, LLC, Novosibirsk, Russia; 5 SeqLL, Inc., Woburn, MA USA; 6 Oregon Health and Science University, Portland, OR USA

**Keywords:** Attention-deficit/hyperactivity disorder (ADHD), RNA sequencing, Transcriptome, GIT1, Galactose, Twins

## Abstract

**Background:**

A variety of DNA-based methods have been applied to identify genetic markers of attention deficit hyperactivity disorder (ADHD), but the connection to RNA-based gene expression has not been fully exploited.

**Methods:**

Using well defined cohorts of discordant, monozygotic twins from the Michigan State University Twin Registry, and case-controlled ADHD cases in adolescents, the present studies utilized advanced single molecule RNA sequencing to identify expressed changes in whole blood RNA in ADHD. Multiple analytical strategies were employed to narrow differentially expressed RNA targets to a small set of potential biomarkers of ADHD.

**Results:**

RNA markers common to both the discordant twin study and case-controlled subjects further narrowed the putative targets, some of which had been previously associated with ADHD at the DNA level. The potential role of several differentially expressed genes, including ABCB5, RGS2, GAK, GIT1 and 3 members of the galactose metabolism pathway (GALE, GALT, GALK1) are substantiated by prior associations to ADHD and by established mechanistic connections to molecular pathways relevant to ADHD and behavioral control.

**Conclusions:**

The convergence of DNA, RNA, and metabolic data suggests these may be promising targets for diagnostics and therapeutics in ADHD.

## Background

Attention deficit hyperactivity disorder (ADHD) is the most common psychiatric disorder in childhood and adolescence, affecting roughly 5% of youth worldwide [1]. Although heritability is substantial [2], environmental modulation is well known and environmental influences are of keen interest. Further, ADHD is a complex disease, with no single gene showing an overwhelming effect, except in very rare cases [2]. Further, ADHD is likely to be etiologically complex and to reflect multiple underlying etiology types [3]. Thus, ADHD is potentially a collection of disorders, which could involve inherited DNA variations, somatic epigenetic changes during neural differentiation, and environmental modifiers. A variety of genetic technologies have been applied to help identify either biomarkers or clues to causative mechanism, including GWAS, exome sequencing, and others [4].

Physiologically, numerous avenues have been examined for a potential role in ADHD. One theory holds that ADHD involves changes in the glutaminergic neurotransmitter systems, a major excitatory pathway in the brain, however genome-wide SNP analysis has so far failed to identify glutamate receptor DNA variants as a significant association with ADHD [5]. Several lines of evidence, including the utility of drugs such as methyphenidate, amphetamines, and atomoxetine has suggested that deficits in the dopaminergic and adrenergic systems could underlie the neurochemical basis of ADHD [6].

While a major focus has been on neurodevelopmental pathways, there are reasons to be cognizant of potentially important changes in non-neural endocrine systems. The strong linkage of ADHD with male gender has raised potential connections to steroid hormones, but it is difficult to exclude sex-linked genetic elements, and/or cultural perceptions related to the diagnosis of ADHD [7].

It has recently been suggested that RNA sequencing may be informative for identifying ADHD-related biomarkers, based on positive findings in a small paired analysis in 3 multiplex cases [8]. Additionally, targeted and candidate gene studies have stimulated interest in miRNAs in ADHD [9]. The present studies employed a broad and relatively unbiased strategy to obtain exploratory data for hypothesis generation using two types of ADHD cohorts analyzed by high-precision, genome-wide single molecule RNA sequencing.

The present studies employed cutting-edge RNA sequencing technology to very accurately quantify transcript levels across the entire transcriptome, for both coding, and non-coding transcripts. The RNA profiling approach was applied to stabilized whole blood samples drawn from two well-characterized cohorts: (a) children matched on ADHD and non-ADHD status and, (b) identical twins that were discordant for ADHD traits. The results provide a deep and broad map of RNA changes potentially related to ADHD, and can be combined with other ‘omic data, such as genome-wide association studies (GWAS), DNA methylation, and proteomic analysis to help identify new biomarkers, and secondarily, new clues to biological processes related to ADHD.

## Methods

### Participants

#### Non-twin case-controls

Case-controls were selected from the Oregon ADHD Longitudinal Study Cohort (for illustrative prior papers see [10, 11]. For that cohort, families were recruited for a case–control study of ADHD and non-ADHD, by soliciting community volunteers with public advertisements and mass mailings using commercial mailing lists. The local Institutional Review Board approved the studies. Parents provided written informed consent for their minor children under the age of 16; minor children provided written informed assent. All families completed a full multi-informant, multi-method screening process to establish eligibility and diagnostic group assignment for ADHD, non-ADHD, as well as comorbid disorders.

After screening, the research team conducted a diagnostic evaluation using standardized, well-normed rating scales from parents and teachers, parent semi-structured clinical interview, child intellectual testing, and clinical observation. Best-estimate research diagnoses and final eligibility were established by a team of two experienced clinicians (a child psychiatrist and a child psychologist), who independently assigned final diagnoses and comorbid disorders including ADHD, oppositional defiant disorder (ODD), and any lifetime mood disorder (major depression, dysthymia, or other), as reported herein. History of seizures or head injury, parent–teacher rating discrepancy making diagnosis uncertain, psychosis, mania, current major depressive episode, Tourette’s syndrome, autism, and estimated IQ < 80 were a basis for exclusion in the present study.

From 2144 volunteers, 850 eligible children were identified. The group used in the current study were a subset selected because they (a) clearly met Diagnostic and Statistical Manual of Mental Disorders-5 (DSM-5) criteria for ADHD or non-ADHD comparison group (rather than subthreshold), (b) had no prior history of psychiatric medication, (c) were Tanner stage 1 or 2 by parent report on the Pubertal Development Questionnaire, (d) were not a sibling of another child in the cohort; and, (e) were willing to give a blood sample. This process provided a set of non-twin case-controls (n = 48: 24 ADHD, 24 controls) for whom Paxgene-stabilized, frozen blood samples were submitted for RNA sequencing. After RNA sequencing, 23 ADHD and 21 case controls were available, as described in Table 1.

**Table 1:** Demographic and clinical characteristics of subjects

	Non-Twin case controls	Discordant twins
N	n = 48: 23 ADHD, 21 controls	n = 32^a^ (16 pairs)
Age in years (mean, SD)	10.63 (2.0)	12.29 (4.0)
Age (range)	7.47–14.8	8.08–17.86
% male	100%	75%
ADHD-RS T score “ADHD” (mean, SD)	65.6 (9)	62 (9)
ADHD RS T score “non-ADHD” (mean, SD)	43 (5.8)	47 (6.8)
% ever any mood disorder	12.5%	27%^b^
% ever any anxiety disorder	18.75%	30%
% current mood	2.2%	7%
% current anxiety	13%	27%
Comorbid dx at year of blood draw	2.2% mood; 13% anxiety	7% mood; 27% anxiety^b^

^a^2 twin pairs (n = 4) scored very low on discordance ranking scale

^b^Mood and Anxiety reported for one or both twins. Based on 15 pairs, n = 30 (1 pair missing data, n = 2).

#### Twins

Twins were recruited from the Michigan State University Twin Registry (MSUTR; [12, 13]. The registry has over 30,000 twin pairs between the ages of 3 and 55. For the current study, recruitment was carried out via anonymous mailings to all MSUTR, identical (monozygotic, MZ) twin families with twins age 7–17 years old. Volunteering families were then screened for eligibility. They were restricted for the current study to (a) never-medicated youth, (b) age 7–17 (it proved impractical to restrict to pre-pubertal due to the relative rarity of discordant pairs), (c) no major medical illness, autism, or neurological condition in the screen record (later confirmed by clinical interview), (d) believed to be MZ (later confirmed by genotyping), (e) eligible for outreach (limited to accommodate multiple studies accessing the registry).

Those who responded then completed a phone screen for eligibility and, if eligible, were scheduled for a home visit. At the home visit, a trained staff member completed a clinical interview, drew blood into an RNA Paxgene stabilizer tube, and collected saliva into an Oragene tube for DNA isolation. Self- and parent-report rating scales were also collected, as described below.

The number of discordant pairs was limited by two major factors (a) about 1/3 of twin births are monozygotic, (b) ADHD has high heritability, so discordant pairs are rare. The final samples available for statistical analysis after clinical screening, RNA sequencing, and data quality checks is shown in Table 1, with additional recruitment details provided in Additional file 1: Data S1. After RNA sequencing, there were 16 discordant pairs meeting quality control metrics for analysis. In both cohorts, the size of the final groups was determined by the number of available samples meeting inclusion/exclusion criteria, and then by successful RNAseq analysis.

### Measures

#### ADHD evaluation

ADHD was evaluated by the following measures: parent completed the Conners' Rating Scales-3rd Edition short form [14]; Strengths and Difficulties Questionnaire long form including the impairment module (SDQ) [15]; the ADHD Rating Scale (ADHD-RS) [16], and a semi-structured clinical interview (Kiddie Schedule for Affective Disorders and Schizophrenia, KSADS) administered by a Master’s-degree level clinician trained to reliability with an outside trainer (EB) [17]. For the twin data, these data were used to evaluate consensus ADHD status by two of the authors (JN,EB) blind to the RNA data. For the case–control data, teacher ratings were also available and consensus diagnosis was arrived at by an independent clinical team as described in the online appendix.

#### Definition of discordance

Twins were considered discordant if they met the following criteria:

- At least a 3-symptom separation on the ADHD-RS or KSADS, with one twin below diagnostic threshold and never identified with ADHD; or
- At least a 10 point T score difference on the ADHD-RS, or
- One was previously diagnosed and treated and the other was not (and the untreated twin did not meet ADHD criteria by our measures), or
- Any combination of these criteria.

#### ADHD discrepancy score

To create a quantitative estimate of degree of discordance, the following four variables were created:

- Absolute difference between higher and lower scoring twin on an ADHD–RS (by parent) raw score (range 0–27) obtained at the same time as the RNA blood tube was collected, and rated over the past 6 months for (i) inattention, (ii) hyperactivity-impulsivity, and (iii) total. The total is reported here.
- For the fourth variable, each twin pair was ranked on “degree of discordance” based on considering all available data including multiple time points rating scales (Conners, ADHD-RS, SDQ), the KSADs, and comorbid and substance use conditions. This was achieved by consensus rankings by JN and EB blind to the RNA data.

#### Whole blood RNA isolation

For complete methods, see Additional file 1: Data S1. Whole blood RNA was isolated from the Paxgene blood collection tube using the Paxgene Blood Isolation kit and the Qiagen QIACube Automation System. The RNA was quantified by optical absorbance at 260 and 280 nm using the NanoDrop 1000. The resulting nucleic acids were treated with TurboDNAse to remove residual DNA and then depleted of ribosomal RNA (rRNA) using the Illumina Ribo-Zero rRNA Removal Kit (H/M/R). RNA concentration was measured using the absorbance at 280 and 260 nm (Nanodrop).

#### Single molecule sequencing

First strand cDNA synthesis was carried out with 50–100 ng RNA at 95 °C for 5 min using 50 ng/µL random hexamers (Invitrogen #51,709) and 1 µL of 10 mM dNTP mix (Invitrogen #Y02256). 3′ Poly A tailing was achieved with terminal transferase (NEB #M0315). Cleaned samples were denatured and hybridized to the poly dT surface of SeqLL flow cells. Samples were sequenced using SeqLL’s True Single Molecule Sequencing (tSMS) technology that allows for RNA sequencing without requiring PCR amplification or library preparation ligation steps.

#### Read alignment and quantification

Data output from the sequencer is in raw short read format (SRF) files. SRF files were processed using the HeliSphere Bioinformatics package, first converting to SMS format for alignment. SMS reads were trimmed for leading T homopolymers and were filtered for reads with a minimal length of 25 bases after trimming. Trimmed reads were aligned to the HG38 human genome (GRCh38) supplemented with the complete ribosomal repeat unit (GenBank Accession U13369.1) using the HeliSphere BASIC analysis pipeline.

#### Statistical analysis of differentially expressed genes

From the aligned reads, a variety of analytical approaches were employed in order to identify differentially expressed genes (DEGs) which passed one or more filtering strategies. To minimize the impact of any one statistical method, the sample set was analyzed by 9 methods, deriving from 5 types of analysis: using the EdgeR Bioconductor package [18, 19] using either dispersion analysis (comEdgeR), or generalized regression analysis (glmEdgeR); with TCC package [20] using either edgeR (tccEdgeR), DESeq [21] (tccDESeq) or DESeq2 [22] (tccDESeq2) methods for differential expression analysis; with voom [23] from limma [24]; and also with baySeq [25] and ALDEx2 [26] packages. To find the most commonly identified DEGs, the results of each analysis were ranked by the resulting *p* value likelihood of a difference between groups adjusted for multiple testing using the Benjamini–Hochberg method. To constrain the size of the DEG list, it was predetermined to select the top 100 from each method and then combine them to achieve a single ranked list across methods. These were then ranked by the number of times a given DEG appeared in each of the 9 lists.

**Fig. 1:**
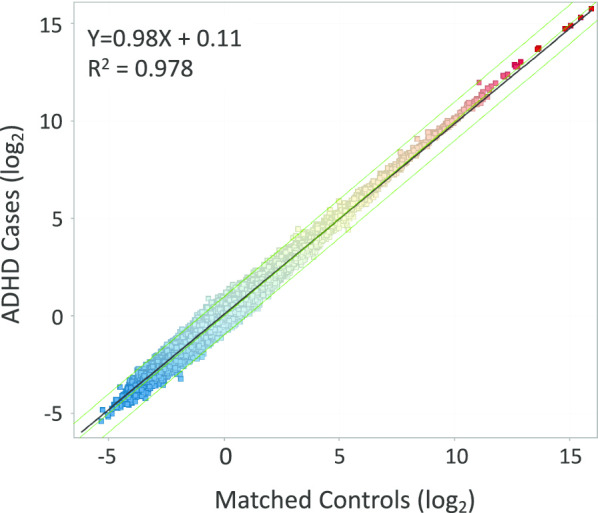
RNA sequencing of whole blood from ADHD Cases versus Matched Controls. Stabilized whole blood was sequenced, aligned, and then counted into UCSC-defined transcripts, expressed as reads per K of transcript per M of total assigned reads per subject (RPKM). Transcripts were filtered for > 0.01 RPKM in 70% of samples in at least 1 of the 2 groups, resulting in 95,511 transcripts remaining. The RPKM level of each transcript (colored squares) is expressed as the mean in Controls (X axis) versus the mean in ADHD cases (Y axis) for each of the transcripts, and plotted on log2 scale. The central green line is a slope of 1, bounded on each side by twofold change lines. Black line is the actual fit of the data distribution, defined by the equation in the upper left

A second general strategy that has proven useful, due its simplicity and absence of assumptions about the distribution of RNAseq data, is to adjust the transcript counts only by correction for the transcript size and total informative reads. The RPKM calculation compensates for the size of the transcript, and for the total number of reads acquired. The number of reads aligned to each transcript are divided by the size of transcript in thousands of base pairs (per K), and then divided by the total number of informative reads obtained for that subject (in Millions), yielding RPKM. To further constrain the DEG list to transcripts with detectable expression, the total aligned read counts for the HG38 genome (GRCh38, n = 195,187) were filtered to include transcripts present at > 0.01 RPKM in 70% of the samples of at least one diagnostic group, leaving ~ 95 K working transcripts for analysis (see Fig. 1 for scatter plot of RPKM expression per transcript).

## Results

### Clinical parameters of the study subjects

#### Case controlled cohort

A total of 100 never-medicated children on whom Paxgene stabilized blood was available were used to compose 24 pairs of ADHD affected or unaffected case controls. They were selected so that each ADHD case was matched by age and gender to an unaffected control. From these 24 pairs, 23 ADHD and 21 controls were successfully RNA sequenced to pre-specified criteria of read depth. The characteristics of the groups are shown in Table 1.

#### Discordant twins cohort

An initial cohort of 50 never-medicated, potentially discordant identical twin pairs was identified and then narrowed for clinical criteria focusing on a high degree of discordance in the severity of ADHD symptoms. A set of 24 pairs of twins with strong to moderate discordance were identified and 16 pairs had sufficient RNA quality and yield, and successful RNAseq data for further analysis (Table 1).

### Analysis of differentially expressed genes

#### Case controlled cohort

The average yield of nucleic acids was 5.4 ug/tube with an average 260/280 ratio of 2.3 and a BioAnalyzer RIN RNA quality index of 8.97. The tSMS method produced a very broad profiling of ribosome-depleted RNA transcripts in stabilized whole blood. By averaging all subjects in each group, filtering out low expressing transcripts of < 0.01 RPKM, and comparing average RPKM for each transcript between groups, it is observed that tSMS yielded linear quantification (slope = 0.98) of gene expression over ~ 22 log2 orders of magnitude for more than 95,511 transcripts (Fig. 1).

**Fig. 2:**
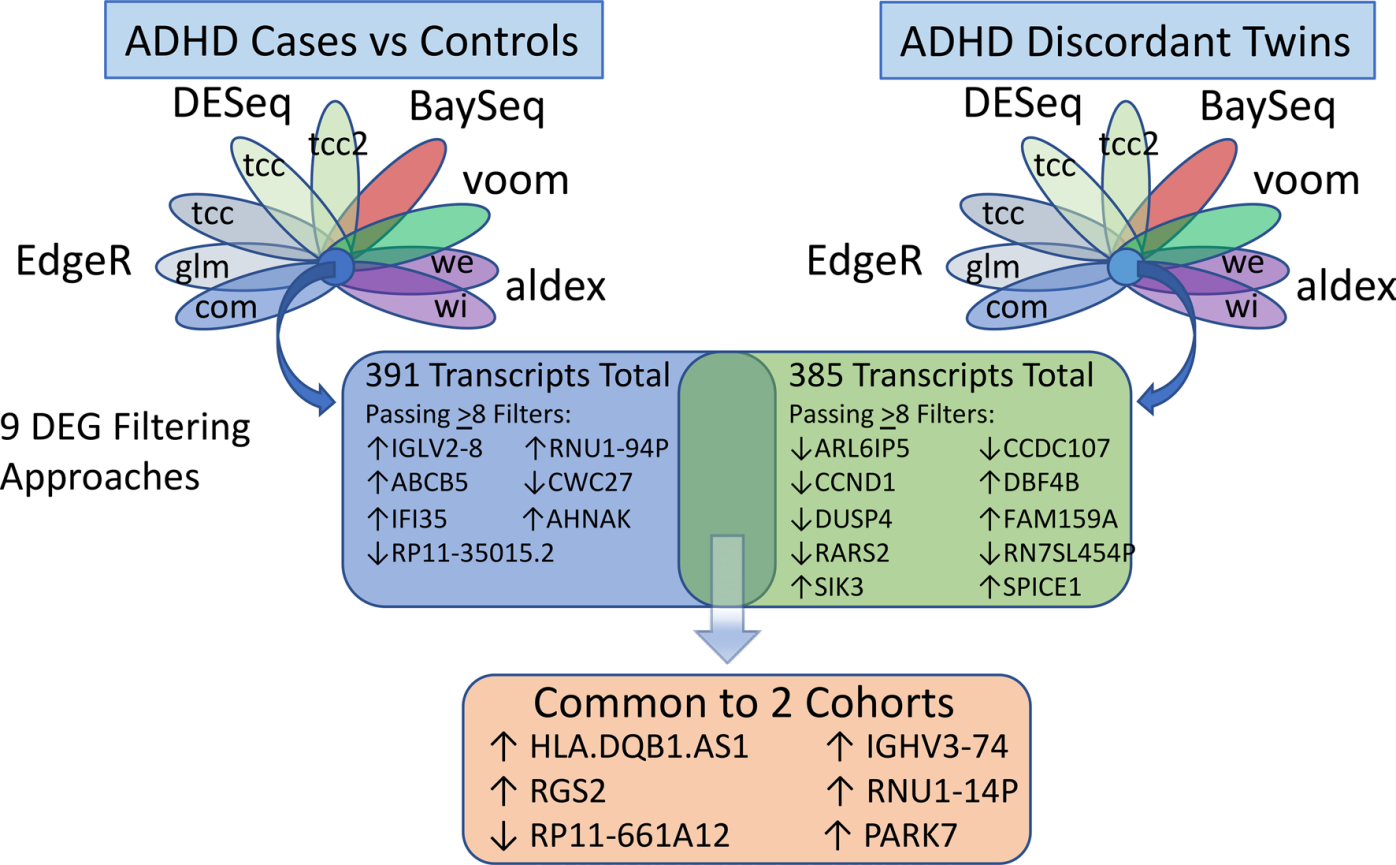
Schematic representation of analytical strategy and top hits. Two separate cohorts: ADHD Cases vs Control and ADHD Discordant Monozygotic Twins were analyzed for whole blood RNA levels by RNAseq. The data was analyzed by 9 distinct methods (i.e. EdgeR/com/glm/tcc, DESeq, etc.) and then differentially expressed genes (DEG) identified by multiple methods resulted in 391 transcripts for Case/Control of which 7 passed 8 or more filters. For discordant twins, 385 transcripts were identified of which 10 passed 8 or more filters. Comparing the 393 and 385 lists identified 5 transcripts identical in both cohorts

Case Controlled Differentially expressed genes (DEGs). Employing the 9 filters approach, 391 transcripts were ranked in the top 100 by at least one method. Of these, two transcripts were identified by all 9 methods, and 5 transcripts were identified by 8 of the 9 methods. Note that although this reduces the likelihood that their differential expression is dependent on only one analytical approach, it does not fully eliminate potential type I error because these are not completely independent methods (Fig. 2). The full 391 gene list by *p* value in the 9 methods can be found in Additional file 2: Table S1. Jaccard similarity analysis reveals the best concordance between methods edgeR, DESeq and DESeq2. The most discordant results were obtained with BaySeq (Additional file 3: Data S2). The 2 transcripts passing all 9 methods with a corrected p < 0.05 are difficult to interpret: IGLV2-8 (↑2.0X in ADHD) is an immunoglobin lambda chain V-II region, and its expression appeared related to at least 10 other DEGs that were either HLA or IgG-related. More difficult to interpret is RNU1-94P (↑2.0X), which is a small nuclear protein pseudogene. The five transcripts passing 8 filters included 4 with better annotation: ABCB5, CWC27, IFI35, and AHNAK:

ABCB5 (↑1.9X in ADHD) is a member of the multi-drug resistance (MDR) family of transporters. ABCB5 has been mechanistically linked to glucose, phospholipid, and amino acid transport [27], and copy number variants have been linked to childhood obesity [28]. ADHD is also associated with increased risk of obesity, with mechanisms unknown [29].

CWC27 (↓1.4X) is a spliceosomal trans-peptidyl-isomerase that has been associated with retinal abnormalities and developmental disorders, including neurological defects, in children with mutations [30].

IFI35 (↑1.4X) is an interferon-induced protein that acts as a ‘damage-associated molecular pattern (DAMP)’ [31], and thus could indicate some type of inflammatory source in ADHD. Substantial circumstantial data suggests inflammation may play a role in ADHD [32].

AHNAK (↑1.3X) is a particularly interesting target because it is a giant 680 kD neuroblast differentiation-associated protein, that has been associated with a range of relevant neurological disorders including bipolar disorder [33], depressive-like behaviors in knockout mice [34], ß-adrenergic regulation of the cardiac CaV1.2 calcium channel [35], and a variety of immune functions. The AHNAK family member, AHNAK2 (↑1.9X), is also identified on this list, with DEG identification by 2 of the analytic methods.

The fifth transcript, RP11-35015.2 (↓1.6X) is a poorly annotated transcript that lies within intron 1 of the IGF1 receptor (IGF1R), and thus, difficult to more clearly understand.

The complete list of 391 selected (Additional file 2: Table S1) includes other interesting transcripts, such as BACE2 (↓1.6X) and MED6 (↑1.5X). However, we proceeded to further narrow this case–control list by virtue of analyzing the cohort of discordant twins, and then determining whether any systematic patterns of similarity emerged.

### Discordant twins cohort

tSMS of 16 discordant twin pairs produced transcript profiling of similar breadth and linearity as observed in the case–control study (Additional file 4: Fig. S1). Using an essentially identical analytical approach to the case controls (Fig. 2), the results of RNAseq from monozygotic ADHD-discordant twins were subjected to 9 analytical approaches and then the top 100 transcripts from each were ranked by their presence on the 9 lists. The resulting list of 385 transcripts can be found in Additional file 5: Table S2.

Discordant twins DEGs: A total of 10 transcripts passed 8 of 9 filters and contains transcripts with close similarity to several of the case–control DEGs (Fig. 2). These high-ranking transcripts present potential hypotheses for further study with regard to ADHD, as follows:

ARL6IP5 (↓1.4X) is an ADP ribosylation factor-like GTPase 6-interacting protein, but is also known as JWA, a homologous gene of the glutamate-transporter-associated protein 3–18 (GTRAP3-18), and addicsin. ARL6IP5/JWA is expressed at high levels in the hippocampus and ARL6IP5/JWA knockout mice showed spatial cognitive potentiation and enhanced neurite growth in newborns [36]. Conditional astrocytic ARL6IP5/JWA null mice demonstrates a role as a neuroprotective factor against dopaminergic neuronal degeneration [37]. ARL6IP5/JWA has been associated with increased expression in the amygdala after chronic morphine treatment [38], and with morphine dependence via the delta opioid receptor [39].

CCDC107 (↓1.9X) is closely related to CCDC132 and CCDC84 found in the case control study. While relatively little is known about these coiled coil family members, coiled coil helix proteins (e.g. Chchd2) have been implicated in ADHD-like mouse models [40].

CCND1, cyclin D1 (↓2.9X), is related to CCNC and CCNL2 from the case control studies. While the cyclins are largely studied in relationship to cell cycle control, they can serve a variety of regulatory functions in cells.

DBF4B, DBF4 Zinc Finger B (↑2.5X), has been extensively studied as an activator of the Mcm2-7 helicase, a partner to Cdc7 kinase, and thus important for the initiation of DNA replication. Potentially of interest, it has also been associated with autism spectrum disorders via a semaphorin 5A (SEMA5A) eQTL network [41].

Dual specificity phosphatase 4 (DUSP4, ↓3.2X) is a family member to DUSP6 from the case–control study, and has recently been described as a control element in the suprachiasmatic clock network via modulation of vasoactive intestinal peptide signaling to ERK1/2 [42].

FAM159A (↑1.7X) has counterparts FAM104A, FAM134B, FAM157C, FAM162A, and FAM213B as differentially expressed in the case–control study. Little is known about FAM159A, but FAM134B, aka RETREG1 reticulophagy regulator 1, has a substantial literature connecting it with various functions including autophagy, and sensory neuropathy in humans [43] and Border Collies [44]. Inhibition of FAM134A causes impaired proteostasis in the endoplasmic reticulum due to the accumulation of misfolded proteins, which has been implicated in vascular dementia [45]. FAM162A is associated by GWAS to a gene-by-alcohol dependence interaction study of risky sexual behaviors and so it could be related to behavioral control [46]. Coincidentally, ADHD is associated with increased sexual risk taking [47].

RARS2 (↓1.6X) is the arginyl-tRNA synthetase gene that has been associated with a spectrum of neurological disorders including myoclonic epilepsy, mental retardation, spasticity, and extrapyramidal features [48]. Patients with RARS2 mutations exhibit early onset hypotonia, epileptic seizures, encephalopathy, and feeding difficulties in a syndrome termed pontocerebellar hypoplasia type 6 (PCH6) [49].

RN7SL454P (↓1.95X), has counterparts RN7SL423P and RN7SL687P as DEG in the case control cohort. It appears to be a small non-coding transcript, intronic to the dynein axonemal heavy chain 17 gene on chromosome 17 (DNAH17), but with no known function.

SIK3.IT1 (↑2.1X) is salt-inducible kinase 3, with known relations to sleep and circadian rhythm, and to glucose and lipid homeostasis, steroidogenesis, and adipogenesis [50].

SPICE1(↑1.4X) is a spindle and centriole-associated protein, which might relate to DBF4B and CCND1 in regards to cell cycle control. Computational screening identifies it as an aurora kinase substrate and it is known to cooperate with CEP120 in centriole elongation. Interestingly, SIK3 also interacts with aurora A, aurora B, and polo-like kinases, and SIK3 repression enhances the antimitotic effect of aurora inhibition [50]. Likewise, CCND1 has known interactions with aurora kinases [51]. The coiled coil proteins, potentially including CCDC107, are commonly associated with the centrosome maturation and aurora kinases [42], suggesting several possible coregulatory scenarios for SPICE1, CCND1, DBF4B, and SIK3 in ADHD, potentially in a non-mitotic, but centriolar/aurora kinase-mediated control of gene expression.

#### DEGs common to both cohorts

From the 385 DEG list compiled from 9 analyses of the discordant twins (Additional file 5: Table S2), 6 transcripts are identical to the 391 DEG case–control results obtained by similar methods (Additional file 2: Table S1). While 6 identical matches between 2 different cohorts of ADHD subjects could occur by random chance (Fisher’s exact text *p* = 0.318), it does suggest that these transcripts may merit further analysis as hypotheses in future studies.

HLA.DQB1.AS1, as the name implies, is an antisense transcript to the HLA-DQB1 locus on chromosome 6, which is elevated about twofold in ADHD cases. As noted previously, particularly in the case–control study, a substantial group of transcripts were HLA or IgG related, implying that some type of immune defect is at work. Because whole blood is being profiled, one must be cautious about an over-representation of immune-related transcripts (which are very plentiful in whole blood), but conversely, one cannot dismiss immune involvement as noted earlier. The potential role of inflammatory factors in ADHD has been raised over the years and is supported by various circumstantial data as recently reviewed [32], and recent analysis suggests the potential role of HLA loci in neurodevelopmental disorders such as ASD, and to a lesser degree ADHD [52].

In the same vein, IGHV3-74 is the variable region heavy chain transcript involved in antigen recognition by encoding IgM antibodies. While speculative, increased levels in the 2 cohorts could suggest some type of immune or autoimmune activity in ADHD.

The regulator of G protein signaling RGS2 is increased in both cohorts. RGS2 has diverse actions including promoting the translation of stress-associated proteins ATF4 and CHOP via an eIF-2B inhibitory domain [53]. Of potential importance, RGS2 variants have been associated with childhood adversities as predictors of anxious and depressive responses [54], as well as the regulation of nicotine-induced anxiolytic activity in mice, and cocaine-induced rewarding effects [55, 56]. Likewise, RGS2 is thought to mediate the anxiolytic effects of oxytocin [57], and affects T cell activation, anxiety, and male aggressive behavior [58]. RGS2 knockout mice exhibit increased fear learning, spatial learning, and neophobia [59]. Further, RGS2 modulates the activity and internalization of the dopamine D2 receptor in neuroblastoma cells [60], and has been implicated in dopamine receptor signaling during amphetamine self-administration [61].

Of potential interest is Park7 RNA (DJ-1), which is extensively investigated as related to early onset Parkinson’s Disease [62]. Based on some symptomatic similarities between Parkinson’s and ADHD, especially impulsivity [63], it was suggested there may be shared underlying causative factors. However, the circulating plasma protein levels of Park7 were not associated with ADHD in 125 ADHD patients versus 66 healthy controls [64], although whole blood RNA levels were not assessed.

Changes in RNU1-14P, which is a small nuclear pseudogene, is quite difficult to interpret, as is the RP11-661A12 transcript, though the latter is potentially an upstream ORF or alternate 5′ exon for the zinc finger CCCH-type containing 3 (ZC3H3) transcript, which is involved in nuclear adenylation and export of mRNAs [65].

#### Pathway analysis

**Fig. 3:**
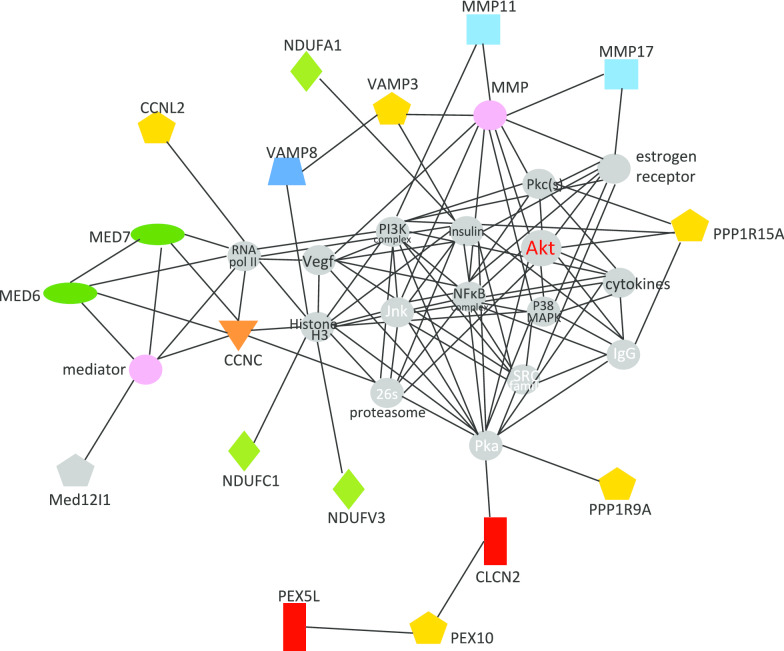
Pathway analysis of differentially expressed genes common to twins and case–control studies: AKT Pathway. Differentially expressed transcripts identified by both the case–control and twin studies were compared to a pre-curated database of biological pathways to determine whether any pathways were disproportionately affected. The top ranking hit, the AKT pathway, is shown schematically with gene products as polygons connected by lines indicating their known relationships. Transcripts identified in the present analysis are shown in color, with other transcripts in the pathway shown in gray

**Fig. 4:**
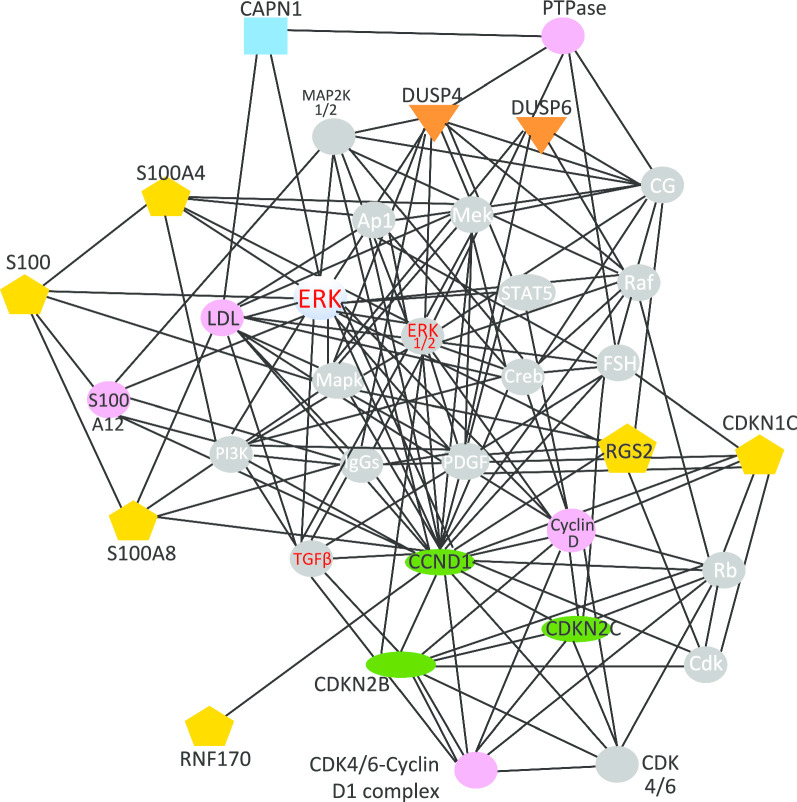
Pathway analysis of differentially expressed genes common to twins and case-controls: Erk Pathway. Differentially expressed transcripts identified by both the case–control and twin studies were compared to a pre-curated database of biological pathways to determine whether any pathways were disproportionately affected. The top ranking hit, the AKT pathway is shown schematically with gene products as polygons connected by lines indicating their known relationships. Transcripts identified in the present analysis are shown in color, with other transcripts in the pathway are shown in gray

An additional set of transcripts had very similar isoforms reported in the case control results, for example, MED7 vs MED6, CLIC2 vs CLIC1, JRK vs JRKL. While there is no assurance that these close family members perform similar functions, it is worth considering whether a similar pattern is reflected. A list of 66 of these overlapping transcripts was submitted for an unbiased analysis using pre-curated relationships between the gene products (Ingenuity Pathway Analysis). Several plausible relationships are identified in a manner that could identify latent variables that might account for a substantial subset of the transcript variations. Statistically, the top pathway identified centered around the well-characterized Akt/Insulin/PI3kinase/NfKB axis, as shown in Fig. 3. Underlying changes in glucose to insulin signaling could drive broader changes into the MED6/MED7/CCNC pathway as well as VAMP8/VAMP3/MMP/NDUF pathway. A second, and related, high scoring pathway is the Erk pathway, which would explain the CCND1/CDKN2B/CDKN2C/CDKN1C/RGS2 changes, and also the S100A12/S100A4/S100A8/CAPN1/DUSP4/DUSP6 transcript alterations (Fig. 4).

#### RPKM analysis of the case–control and discordant twin datasets

In the context of a hypothesis-generating, exploratory study such as this, the prior analysis using 9 DEG methods may risk missing biologically important pathways in favor of statistical rigor. The datasets were re-analyzed using an RPKM threshold of 0.01, and combined fold-change (> 1.5) and *p* value (< 0.01, uncorrected) filtering approach that has proven useful in prior biomarker studies [66, 67]. This triple filter identified 524 transcripts in the case–control study (Additional file 6: Table S3), and 505 transcripts in the twin cohort (Additional file 7: Table S4). By filtering for transcripts that were common to both datasets at the gene symbol level, 14 transcripts were identified, but 3 could be excluded because the direction of the changes were in the opposite direction. The remaining 11 transcripts, common by both their presence and direction in both twins and case controls are potentially interesting.

ACP2 (↑2.0X twins) is a lysosomal acid phosphatase that is known to play a vital role in the removal of mannose-6-phosphate residues. ACP2 has known or suspected roles in several neurodevelopmental disorders, as emphasized by mutations in Acp2 causing severe cerebellar and neurodegenerative diseases [68]. Integrated analysis of GWAS and expression data identified ACP2 as a loci related to prepulse inhibition, a measure of sensorimotor gating that is known to be affected in several psychiatric disorders [69].

ALKBH6 (↑2.8X twins) is potentially important because, while relatively little is known about it, by analogy to its homolog ALKBH5, it is likely to function as a methyl-N6-adenosine (m6A) demethylase [70]. While there are extensive investigations into DNA modifications, such as CpG methylation, as a mode of genetic regulation, a quickly escalating literature suggests that defects in RNA modifications are a contributing factor in neurodevelopmental [71], and other disorders [72].

ASPSCR1 (↑2.6X twins), is a UBX domain containing tether for SLC2A4, which has a known fusion protein to TFE3 that is involved in certain cancers. However, better known as TUG, it has important roles as an interactor with the glucose transporter GLUT4, with regulatory activity over insulin-regulated aminopeptidase (IRAP) and vasopressin secretion [73]. While complex, vasopressin has been associated with ADHD by virtue of its known relation to social behaviors, and has been investigated as a potential therapy [74].

CLYBL (↑2.6X twins) is citrate lyase beta-like transcript, which encodes a malate/ß-methylmalate synthase with known effects on Vitamin B12 levels [75]. Vitamin B12 was thought to have a role in ADHD, but supplementation studies have not reported consistent beneficial effects [74]. The role of malate/ß-methylmalate in human physiology is incompletely studied, but methyphenidate treatment in rats causes significant changes in the citrate, malate, and isocitrate synthetic enzyme levels in the brain [76].

**Fig. 5:**
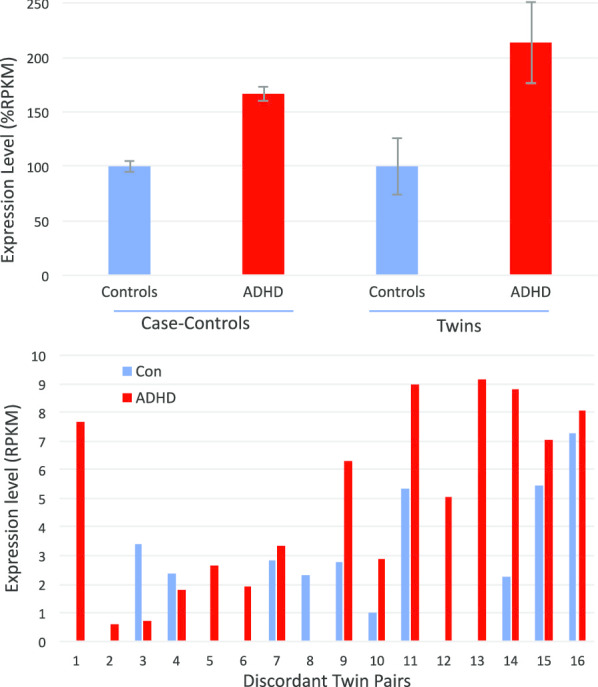
GAK mRNA expression in ADHD case-controls and discordant twins. **a** Average transcript levels between groups in the case–control (n = 22 per group) and the discordant twins (n = 16 per group). mRNA levels expressed on log2 scale. **b** Detailed levels of GAK mRNA expression in the ADHD-discordant, monozygotic twin pairs (RPKM)

GAK (↑2.1X twins), cyclin G associated kinase, is potentially interesting in relation to ADHD. GAK (auxilin-2) has known involvement in synaptic function and neurological diseases [77], and is associated by GWAS with overlapping properties of Parkinson’s Disease and autoimmune diseases [78]. GAK was elevated in both cohorts (Fig. 5a) and in 14/16 of the discordant twin pairs, often in fairly striking fashion (Fig. 5b). GAK mRNA expression across a range of human tissues shows relatively high expression in the cerebellum, about twice the level observed in whole blood (Additional file 4: Fig. S2, GTEX).

**Fig. 6:**
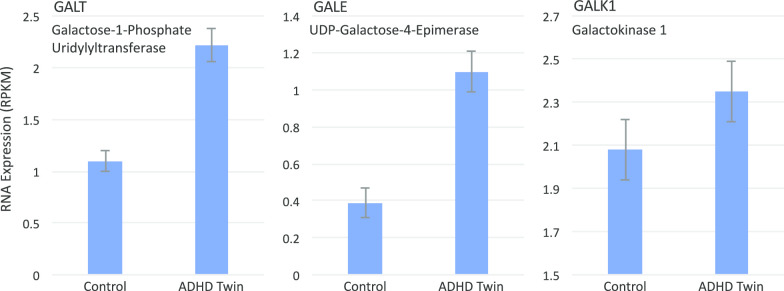
Differentially Expressed Genes in the Galactose Metabolism Pathway. Mean expression level of GALT (uc011lop.2, EC 2.7.7.12), GALE (uc057dhf.1, EC 5.1.3.2), and GALK1 (uc002jpk.4, EC 2.7.1.6) expressed in RPKM are shown for the control, unaffected twin, and the ADHD-affected twin. Bars are mean ± S.E.M

GALE (↑2.9X twins), UDP-galactose-4-epimerase, is one of 3–4 key enzymes in the synthesis and utilization of galactose, and changes in the other members of this family, especially GALT and GALK, were noticeably affected in the ADHD cases, with all 3 of these enzymes in the galactose processing pathway being elevated in the ADHD-affected twins (Fig. 6).

**Fig. 7:**
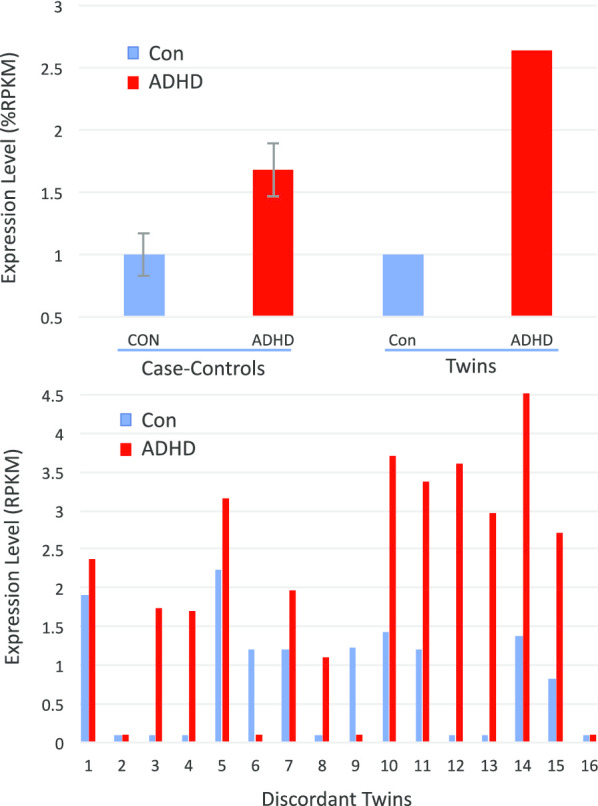
GIT1 in case controls and discordant twins. Upper panel: Average GIT1 mRNA levels by RNAseq in case–control study and the discordant twins study (Uc060djr.1 variant, mean RPKM as % control + S.E.M.). Lower Panel: GIT1 mRNA levels (Uc060djr.1 variant, mean RPKM) in 16 pairs of monozygotic twins discordant for ADHD severity

**Fig. 8:**
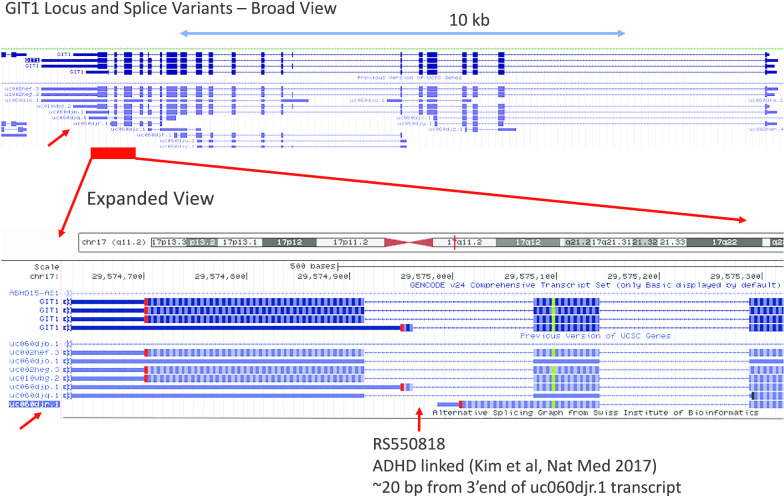
GIT1 Locus. Upper panel: Broad view of the GIT1 locus on 17q11.2 (chr17:29,569,429-29,593,667) showing several of the common splice variants, including the uc060djr.1 variant (red arrow). The region highlighted by red bar is magnified in the lower panel to show the position of the ADHD-linked SNP near the 3′ end of the uc060djr.1 transcript variant

GIT1 (↑2.6X twins) was elevated > twofold in the ADHD subjects in both the discordant twin and case–control cohorts (Fig. 7, upper panel). Several striking coincidences draw attention to GIT1 as potential target. First, of the 15 known GIT1 isoforms, the changes in both cohorts seems largely restricted to a single isoform (uc060djr.1), which was elevated in 12 of 16 discordant twin pairs (Fig. 7, lower panel). GIT1 SNPs were previously associated with ADHD by genome-wide association studies (GWAS) studies that employ a relatively unbiased view of known genetic variation [79], although other cohorts did not support this association [80]. Fine mapping identifies an intronic SNP in GIT1 which causes reduced expression of GIT1 RNA and protein [81]. Strikingly, GIT1 is extensively spliced (Fig. 8), and the intronic SNP localizes to within 20 bp of 3′ terminus of the uc060djr.1 isoform identified in the present RNAseq analysis (Fig. 7). GIT1 knockout mice have ADHD-like traits including a shift in the neuronal excitation/inhibition balance associated with a decreased glial GABA intensity [4], and behavioral correction with methyphenidate and amphetamine [79]. Mechanistically, GIT1 is thought to play an important role in neurite outgrowth [82], synapse formation [83], and the turnover of ß2-adrenergic and other G-protein coupled receptors [84]. GIT1 is expressed at relatively high levels (tenfold above blood) in most brain regions, tibial nerve, and the testes (Additional file 4: Fig. S3, GTEX). While we cannot rule out a type I error, the present data suggests GIT1 merits further consideration as a factor in ADHD.

STAM2 (↑1.5X twins), signal transducing adaptor molecule 2, is a member of the endosome-associated ESCRT-0 complex that is highly expressed in neurons, especially in the cerebral and cerebellar cortex, hippocampus, and medial habenula [85]. STAM2 regulates signaling via Jak2 and Jak3, which are directly involved in c-myc induction of IL-2 [86].

The remaining targets identified in both cohorts are more difficult to interpret. ERCC6L2 (↑2.9X twins) is excision-repair like 2, which has known relevance in cancer, but is difficult to connect with ADHD. HDLBP (↑1.3X twins) is highly relevant to high density lipoprotein metabolism, but has only a tenuous connection to ASD by virtue of a 2q27 deletion that causes reduced expression of HDLBP and 2 other genes [87]. IDS (↑1.4X twins), iduronate 2-sulfatase, is highly studied in Hunter syndrome mucopolysaccharidosis [88], but has no known relation to ADHD. UBE2J2 (↑2.5X twins) directs the ubiquitination of hydroxylated amino acids in the ER, but has no reported connection to ADHD or other developmental disorders [89].

### Correlation between ADHD discordance and gene expression discordance

To further narrow candidate gene expression to potentially important correlates of ADHD, we moved from a categorical to a dimensional analysis of ADHD severity, building on evidence that ADHD functions like a trait in the population [90–92]. As explained earlier, ADHD severity scores (based on parent ADHD-RS raw scores) between the identical twins were compared to create a ‘discrepancy score’ for the twins. These scores were then ranked, with highest discrepancy (most different) being ranked 1, and then correlated to the difference in gene expression (fold change) between the paired twins, for the 505 RPKM list of transcripts (Additional file 7: Table S4). In an ideal scenario, the fold change would inversely correlate to the rank discrepancy (high fold change in gene expression, i.e. 10, associates with lowest numerical rank, ie 1, most discrepant). Negative correlations of r > -0.4 were observed for several transcripts of interest (boxed yellow, Additional file 8: Table S5), and closer inspection suggests they might have potential relevance to ADHD.

Among the highly correlated DEGs, RN7SKP194 (r = -0.60) bears some general similarity to the RN7SL454P target identified by the 9 filter approach, and discussed above. Both of these small nuclear pseudogenes are likely to have as yet unknown regulatory functions [93]. SRP14, signal recognition particle 14, is potentially interesting because it is 5–sevenfold lower in the ADHD twins, and it is part of a larger riboprotein complex thought to regulate translational arrest during protein synthesis in dendrites [94]. GMFG, glia maturation factor gamma, is almost 16-fold lower in the ADHD twins and affects a diverse range of cell types. MICU1, elevated threefold in ADHD twins, encodes a Ca^+2^-sensing, regulatory subunit of the mitochondrial uniporter, and mutations in MICU1 cause a range of symptoms that include progressive extrapyramidal signs, learning disabilities, and fatigue [95]. Among positively correlated transcripts, whereby the most discordant pairs showed the least fold-change in expression was GIT1 with r = 0.585 and an average increase of 2.4 fold in the ADHD twins. Unfortunately, the changes in RNA levels are generally not perfectly correlated with changes in protein expression (r = ~ 0.6) [96], and so this unexpected relationship may not be a significant impediment to GIT1′s relevance to ADHD.

### Comparison to prior genetic studies

Prior exome sequencing of sporadic ADHD cases compared to sibling/parent triads identified ~ 8 interesting targets [4]. Of those, exome mutations in TBC1D9 are a relatively close match to RNAseq expression changes in TBC1D17 observed in the present discordant twin pairs. This suggest a closer look at this family of proteins, likely to be important in vesicle transport, may be warranted. A second possible match is between exome mutations in WDR83, and expression changes in WDR45B in discordant twins, and WDR74 in case control subjects. Also, we observed some similarity to transcripts identified in ADHD by Liao et al. [97], whereby transcripts MED8 and ARTN had suggestive *p* values in our analysis.

## Discussion

Among the strengths of the present approach are the unique and well-characterized ADHD cohorts. In particular, monozygotic, but discordant twins present a powerful genetic model for comparison, and here demonstrated some intriguing similarity in expression pattern with a case–control cohort. They open the possibility of understanding environmental influences while largely controlling for genotype. An important methodological detail is that the RNAseq analysis examines the expression pattern on a relatively high resolution scale to the level of transcript isoforms. Other common RNAseq analytic platforms tend to aggregate expression to ‘gene level’ expression as a single transcript, which has the effect of masking changes in alternatively spliced transcripts. The case of GIT1 is an excellent example of where very specific changes in one splice variant might have high relevance to the disorder in question.

Limitations to the present study principally derive from the observational nature of the studies, a necessarily small samples for MZ discordant twins, and the necessity to use peripheral blood RNA, as opposed to a tissue more proximal to the presumed neural influences on ADHD. Further, the mRNA profiling gives us a very comprehensive view of the transcriptome, albeit at a specific point in time, and without strict control of the mental or physical state of the participants. Of course, causality is indeterminate: we cannot evaluate, for instance, whether changes in the galactose pathway ‘cause’ ADHD, or somehow result from the increased activity or altered diet or other behaviors of the children. A third option, which must be considered, is that both ADHD and galactose changes could result from changes in a different pathway, or from coincidental differences in ADHD teens, such as diet, drugs, or activity. While we can exclude ADHD medications as a source of variations herein, it is difficult to exclude other type of nutritional or nutraceutical differences.

Technically speaking, the RNAseq approach is intrinsically limited by the known genome and transcriptomes that are used to align and interpret the reads. Every RNA profiling method has unique ‘gaps’ and biases that can influence the outcomes, and thus it should not be surprising if a different RNAseq method identified other different differentially expressed transcripts. Further, the RNAseq of whole blood allows for the possibility that the types of cells present in blood at the time of sampling differ from patient to patient, or group to group. Because blood cell counts were not available on the subjects at the time of the blood draw, we were unable to identify such differences or adjust for them. Additionally, a valuable approach to understanding this large data set would be to conduct co-expression analysis, which is a logical next step that might reveal systematic changes not apparent from the current analysis [98–100].

While the present results therefore should be seen as preliminary, the nature of this work is largely unprecedented and therefore it is valuable to note that several patterns were identified that are suggestive as hypotheses for further investigation. Collectively, the results affirm some prior targets, such as GIT1, that were identified by DNA-based technologies, as also relevant to ADHD at the RNA level. Several new pathways are brought to light as potentially productive ground for further exploration. Based on a variety of lines of evidence, it would be quite unlikely if there is a single etiologic cause for ADHD, and the present results demonstrate that none of the RNA transcript changes were observed to occur in all of the youth. It is interesting to speculate that changes in GAK or GIT1, which showed quite strong changes in some, but not all subjects, could indicate particular genetic/epigenetic subtypes of ADHD. The cohort sizes obviously were not powered for a detailed subtype analysis.

An intriguing future direction would explore the possible role of the galactose pathway in ADHD, either as a modulator of core energy sensing via the insulin/AKT/NFkB/mediator pathway (Fig. 3), or as a regulator of galactosylation of key factors in the neurotransmitter pathway, as highlighted by consistent changes in GALE/GALK/GALT. Galactose metabolism could be related to energy sensing and inflammation via the well-established glucose/lactose/galactose connection to the immune/inflammatory pathways that is key to the obesity/insulin resistance/inflammation connection (reviewed in [101]). Additionally, galactose modification to proteins alters their inflammatory potential and is thought to be a key component of ‘inflammaging’ [102]. Potentially the most obvious effect of altered galactose metabolism would be the direct effect on the transport of dopamine to the brain, via galactose modification of dopamine [103]. Dopamine itself is poorly absorbed in the brain, but galactosylated dopamine has increased transit across the blood–brain barrier, and in mouse models increases attention without reducing activity [104]. Given a well-documented relationship between dopaminergic dysfunction and ADHD [105], it is quite plausible that perturbation of the galactose pathway in humans could produce an ADHD-like syndrome.

## Conclusions

These results are the most extensive discordant MZ study of RNAseq expression in ADHD. The results, while preliminary, suggest several interesting hypotheses for further study.

## Supplementary information

**Additional file 1**. Enrollment details.

**Additional file 2**. Transcript IDs by 9 DEG methods for case/control study.

**Additional file 3**. Transcript IDs by 9 DEG methods for discordant twin study.

**Additional file 4**. Case control T-test 524 transcripts.

**Additional file 5**. Discordant twins paired T-test 505 transcripts.

**Additional file 6**. Discordance DEG correlation.

**Additional file 7**. Detailed methods.

**Additional file 8**. DEG Concordance between methods.

## Data Availability

Expression-level de-identified data is available at Gene Expression Omnibus (GEO Accession GSE159104) for any investigators seeking to reproduce the results, or conduct new analysis within the bounds of the IRB-approved work. For the purpose of respecting the privacy of the participants, and their permission to use their samples only for specific research projects, sequence-level data is available through dbGAP (linked in GEO) upon appropriate permissions from Dr. Joel Nigg (niggj@ohsu.edu). The HG38 human genome used for alignment is available via UCSC (https://hgdownload.soe.ucsc.edu/goldenPath/hg38/bigZips/), and the ribosomal sequences are available at Genbank (https://www.ncbi.nlm.nih.gov/nuccore/U13369.1).

## Abbreviations

ADHD: Attention-deficit hyperactivity disorder
DSM-5: Diagnostic and Statistical Manual of Mental Disorders-5
DEG: Differentially expressed genes
GWAS: Genome-wide association studies
KSADS: Kiddie Schedule for Affective Disorders and Schizophrenia
MSUTR: Michigan State University Twin Registry
MZ: Monozygotic
ODD: Oppositional defiant disorder
RNAseq: RNA sequencing
RPKM: Reads per thousand bases of transcript per million transcripts sequenced
SDQ: Strengths and Difficulties Questionnaire
SRF: Short read format
tSMS: True Single Molecule Sequencing
