# Supplementary material for: Biomarker discovery in attention deficit hyperactivity disorder: RNA sequencing of whole blood in discordant twin and case-controlled cohorts
Source: BMC Med Genomics. 2020 Oct 28;13:160. doi: 10.1186/s12920-020-00808-8 (PMC7594430; doi:10.1186/s12920-020-00808-8)
Supplement: Supplementary file 1 — Additional file 1. Enrollment details. [file 12920_2020_808_MOESM1_ESM.docx]

**Additional Recruitment Details:**

Twin recruitment methods: In brief, we obtained all available eligible and qualified twins available in the twin registry (63% response rate to our invitation to volunteers). The registry has over 30,000 twin pairs of whom about 12,000 were in the age range between 3 and 55, and of these 4000 are MZ. For the current study, recruitment was carried out via anonymous mailings as follows. First, all records were screened for families with (a) an MZ twin pair (by zygosity questionnaire, later confirmed by venipuncture blood draw and genotyping), (b) age 7-17 years (c) currently available for outreach (not in another concurrent study)(n=2110). This list was then filtered for (a) met minimal exclusion criteria (no reported major chronic neurological or physical illness or physical disability), (b) at least one of the twins passed a liberal cutoff of T>60 on the CBCL attention scale (n=307, 14.5%)and (b) a group of age and sex matched pairs with both twins T< 60 (n=285). These n=592 received a mailed invitation, of which n=122 were returned as undeliverable. Of the n=470 that received the mailing, n=295 (63%) responded for possible enrollment. After a telephone screen for eligibility and to explain the study requirements, consent was obtained from n=216 (pre-screen discordant 26, concordant 41, unaffected 149). These families then underwent additional normative questionnaires, formal semi-structured clinical interview (KSADSE) with a masters-degree trained clinician (E.B.) who was trained to reliability with the ADHD team at OHSU, and a blood draw into an RNA Paxgene stabilizer tube and saliva collection into an Oragene tube for DNA isolation. and final consensus assignment to ADHD groups by two clinicians (E.B. and J.N.). This yielded 24 discordant, 15 concordant, and 77 unaffected pairs. Only the discordant pairs were used here.” We have also added the following flow chart to the online supplement.


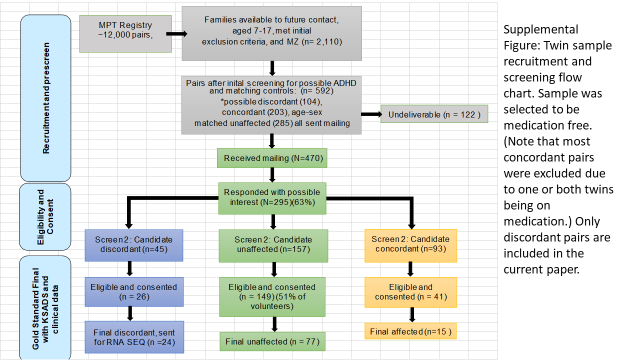


**Additional Technical/Analytical Details:**

**Whole blood RNA isolation.** For complete methods, see Supplementary Data 1. Whole blood RNA was isolated from the Paxgene blood collection tube using the Paxgene Blood Isolation kit and the Qiagen QIACube Automation System. The RNA was quantified by optical absorbance at 260 and 280 nm using the NanoDrop 1000. The resulting nucleic acids were treated with TurboDNAse to remove residual DNA and then depleted of ribosomal RNA (rRNA) using the Illumina Ribo-Zero rRNA Removal Kit (H/M/R). RNA concentration was measured using the absorbance at 280 and 260 nm (Nanodrop).

**Ribosomal RNA depletion.** Ribosomal RNA depletion was performed using the Illumina Ribo-Zero rRNA Removal Kit (H/M/R) (#MRZH11124). Magnetic beads were prepared by vortexing and then aliquoting 225 µL per reaction into RNase-free tubes, placing on magnetic stand, and removing supernatant. Beads were washed twice with RNase-free water, followed by addition of 65 µL of Magnetic Bead Resuspension solution and 1 µL of RiboGuard RNase Inhibitor (as provided by the kit). RNA was treated with rRNA Removal solution by combining ~1-2 µg of total RNA, 4 µL of Ribo-Zero rRNA Reaction Buffer, and 8 µL Ribo-Zero Removal Solution to a total of 40 µL, and incubating at room temperature for 5 minutes. The probe-hybridized RNA solution was then transferred to the previously prepared magnetic beads and the solution was mixed by pipetting, vortexed at high speed, incubated at room temperature for 5 minutes, and subsequently at 50°C for 5 minutes. Tubes were removed from the heat and immediately placed on the magnetic stand. The supernatant, which contained ~90 µL of rRNA depleted sample, was removed to fresh tubes and placed on ice until purification. Ethanol precipitation was chosen as the preferred method of repurification because it provides optimal recovery of small RNAs, such as miRNA and tRNA, as well as mRNA and large noncoding RNA. Volume of samples were adjusted to 180 µL with RNase-free water, followed by addition of 18 µL of 3M sodium acetate, 2 µL of glycogen (10 mg/ml), and 600 µL of 100% ethanol. This solution was gently vortexed and placed at 80°C overnight. After approximately 16 hours, the solution was centrifuged at 10,000 g for 30 minutes and supernatant was carefully removed and discarded. The pellet was washed twice with a 70% ethanol solution, air dried, and dissolved in RNase-free water. Concentration was measured using the absorbance at 280 and 260 nm (Nanodrop).

**cDNA Synthesis.** First strand cDNA synthesis was carried out by initially incubating 7 µL of sample (50-100 ng RNA) at 95°C for 5 minutes and chilling on ice for 2 minutes. While on ice, 5 µL of 50 ng/µL random hexamers (Invitrogen #51709) and 1 µL of 10 mM dNTP mix (Invitrogen #Y02256) were added to the RNA and the mixture was incubated at 65°C for 5 minutes and placed on ice. Subsequently, 4 µL of 5x First Strand buffer and 1 µL of 0.1 M DTT were mixed in to the solution and incubated at 15°C for 20 minutes. Finally, 1 µL of RNaseOUT (Invitrogen #51535) and 1 µL of Superscript III Reverse Transcriptase (Invitrogen #18080-044) were added. The following program was run on the thermal cycler: 25°C for 10 minutes, 40°C for 40 minutes, 55°C for 50 minutes, and 85°C for 5 minutes. Upon completion, 1 µL each of E. coli RNase H (Invitrogen #Y01220) and RNase If (NEB #M0243) were used to digest the RNA strand at 37°C for 30 minutes. EdgeBio Performa DTR Gel filtration cartridges (#42453) were used to purify the cDNA in two successive steps.

**Single molecule sequencing.** 3’ Poly A tailing was initiated by denaturing 10 µL of cDNA plus 3 µL of RNase-free water at 95°C for 5 minutes and snap cooling on ice. A mixture of 2 µL of 10x TdT buffer, 2 µL of 2.5 mM CoCl2, and 2 µL of 1 mM dATP (Enzymatics #N2010) was added to the cDNA, followed by 0.2 µL of 20 U/µL Terminal Transferase (NEB #M0315). The solution was incubated for 30 minutes at 37°C. Finally, the reaction was blocked by spiking with 2 µL of 1 mM ddATP (Roche #12158175103), further incubating at 37°C for 30 minutes, and inactivating the enzyme at 70°C for 10 minutes. Samples were diluted with 1x hybridization buffer, denatured at 95°C for 5 minutes, chilled on ice, and loaded onto the poly dT surface of SeqLL flow cells. Samples were sequenced using script Gen 2.1 30Q 550 FOV with SeqLL true single molecule sequencing (tSMS) technology.

**Read alignment and quantification.** Data output from the sequencer is in raw short read format (SRF) files. SRF files were processed using the HeliSphere Bioinformatics package, first converting to SMS format for alignment. SMS reads were trimmed for leading T homopolymers and were filtered for reads with a minimal length of 25 bases after trimming. Alignments were conducted with indexDPgenomic software, the aligner maximizes the aligned yield of SMS reads due to the ability to align reads in which the predominant error is represented by deletions common in single-molecule sequencing. For the genomic alignments, reads were aligned to the latest NCBI version of the genome supplemented with the complete ribosomal repeat unit (GenBank Accession [U13369.1](https://www.ncbi.nlm.nih.gov/nuccore/U13369.1)) using the HeliSphere BASIC analysis pipeline. The sequence reads were filtered to include reads with a minimal length of 25 bases and aligned using a stringent normalized score of 4.5 (human genome).

**Statistical analysis of differentially expressed genes.** From the aligned reads, a variety of analytical approaches were employed in order to identify differentially expressed genes (DEGs) which passed one or more filtering strategies. To minimize the impact of any one statistical method, the sample set was analyzed by 9 methods, deriving from 5 types of analysis: EdgeR (comEdgeR, glmEdgeR, tccEdgeR), DESeq (tccDESeq, tccDESeq2), BaySeq, voom, and aldex (aldex_we, aldex_wi). To find the most commonly identified DEGs, the results of each analysis were ranked by the resulting p-value likelihood of a difference between groups adjusted for multiple testing using the Benjamini-Hochberg method. To constrain the size of the DEG list, it was predetermined to select the top 100 from each method and then combine them to achieve a single ranked list across methods. These were then ranked by the number of times a given DEG appeared in each of the 9 lists.

A second general strategy that has proved useful due its simplicity and absence of assumptions about the distribution of RNAseq data is to conduct only normalization of the samples by reference to transcript size and total informative reads. The RPKM methods compensates for the size of the transcript, and for the total number of reads acquired. The number of reads aligned to each transcript are divided by the size of transcript in thousands of base pairs (per K), and then divided by the total number of informative reads obtained for that subject (in Millions), yielding RPKM. The total aligned read counts for the HG38 genome (195,187) were filtered for transcripts present at >0.01 RPKM in 70% of the samples of at least one group, leaving ~95K working transcripts for analysis (see Figure 1 for scatter plot of linear distribution).
