# Supplementary material for: Biomarker discovery in attention deficit hyperactivity disorder: RNA sequencing of whole blood in discordant twin and case-controlled cohorts
Source: BMC Med Genomics. 2020 Oct 28;13:160. doi: 10.1186/s12920-020-00808-8 (PMC7594430; doi:10.1186/s12920-020-00808-8)

Supplementary Data 2: Jaccard similarity between 9 DEG methods.

We have examined the correspondence between the 9 analytical methods of DEG analysis. We calculated pairwise Jaccard similarity indices (based on the top100 genes) and obtained the following results: the most correspondence was observed between edgeR, DESeq and DESeq2 variations, the most different results were obtained with BaySeq. The cluster dendrogram comparison is represented below.


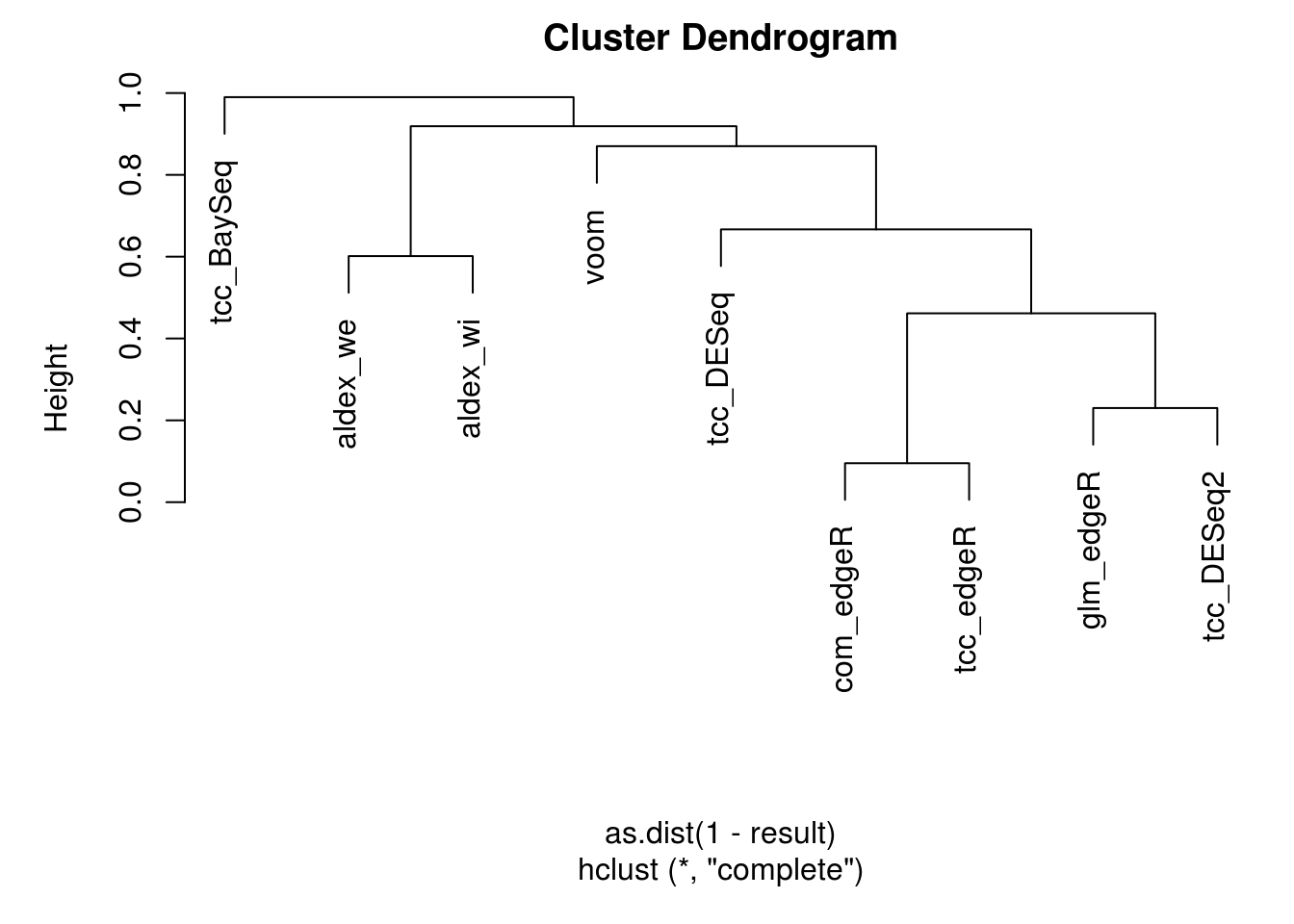

Supplement: Supplementary file 3 — Additional file 3. Transcript IDs by 9 DEG methods for discordant twin study. [file 12920_2020_808_MOESM3_ESM.docx]
