# Supplementary material for: Biomarker discovery in attention deficit hyperactivity disorder: RNA sequencing of whole blood in discordant twin and case-controlled cohorts
Source: BMC Med Genomics. 2020 Oct 28;13:160. doi: 10.1186/s12920-020-00808-8 (PMC7594430; doi:10.1186/s12920-020-00808-8)

Supplementary Figure 1.

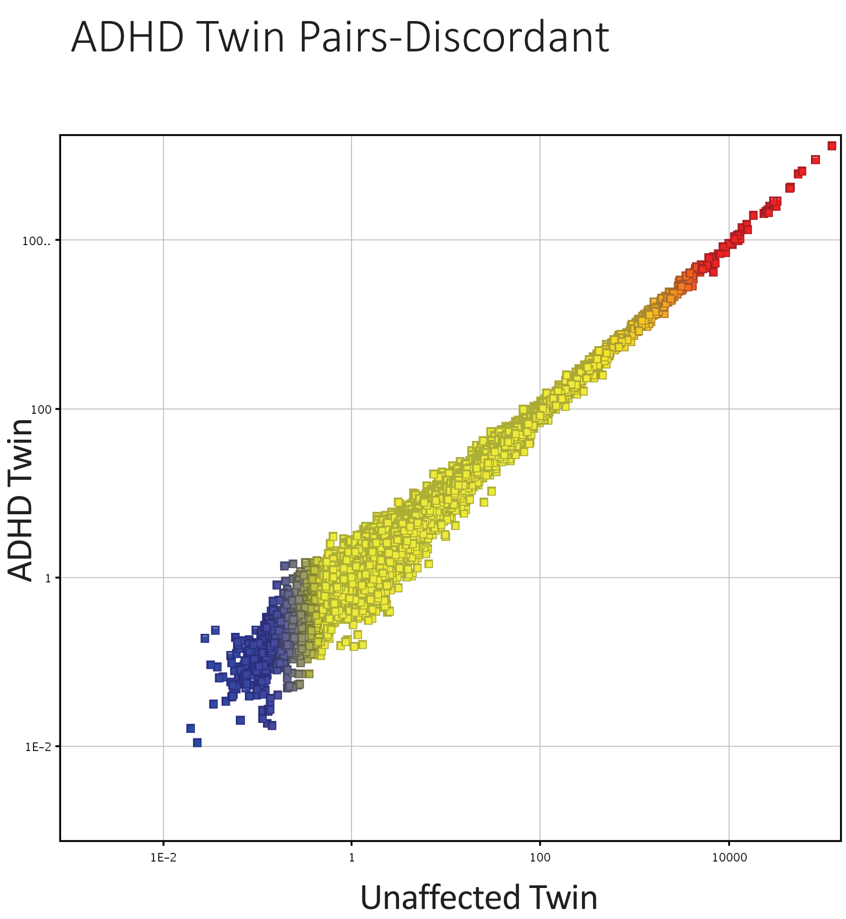

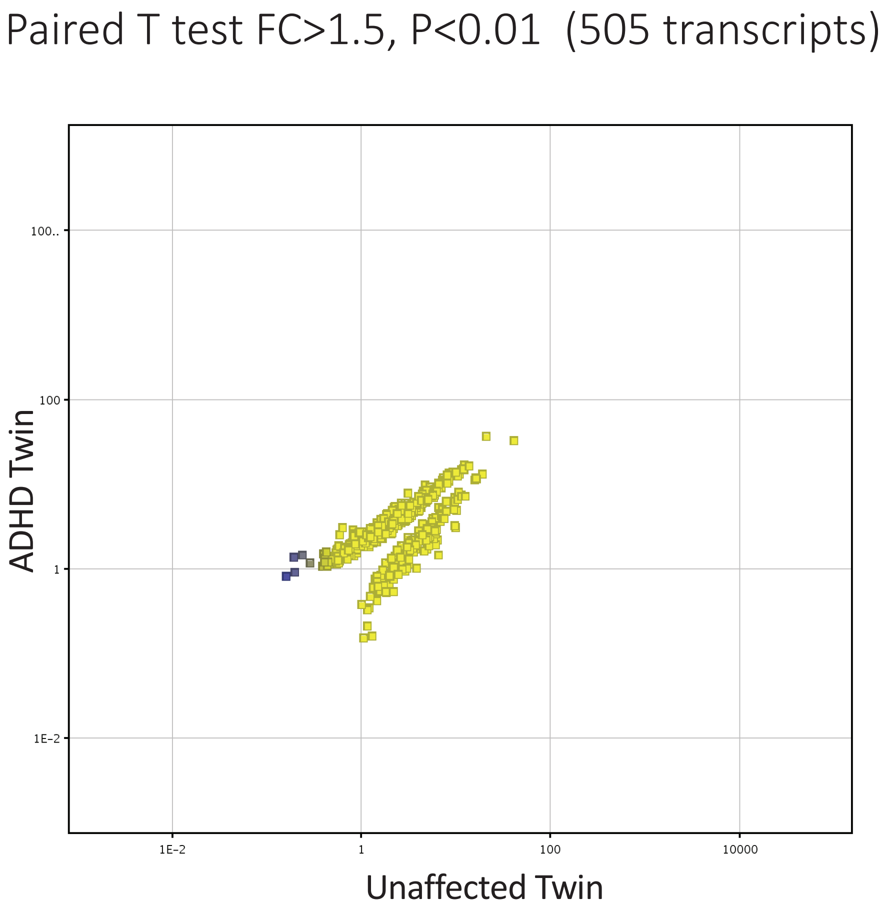

Supplementary Figure 2. GAK Expression in human tissues

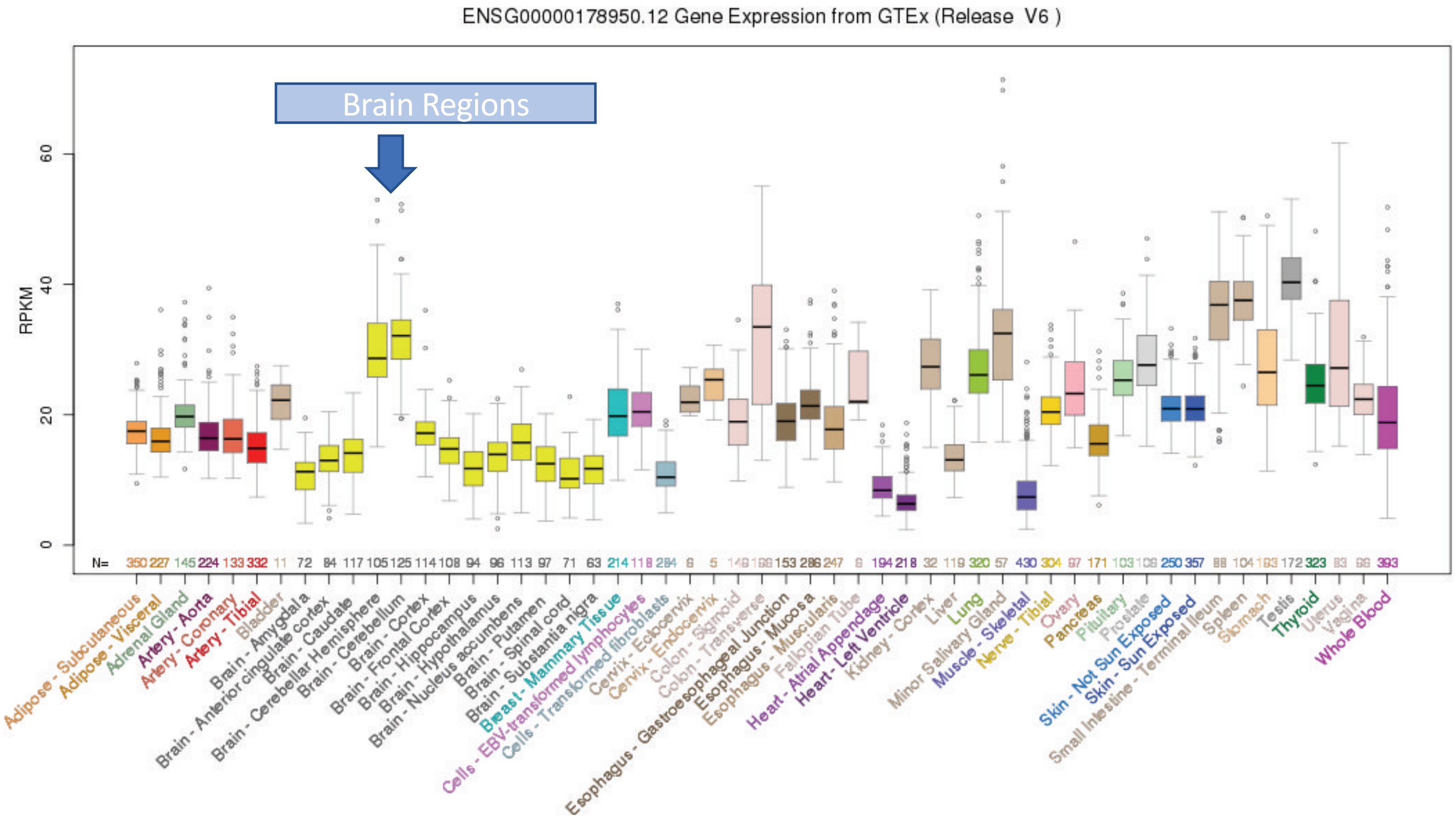

Supplementary Figure 3. GIT1 RNA expression levels in human tissues.

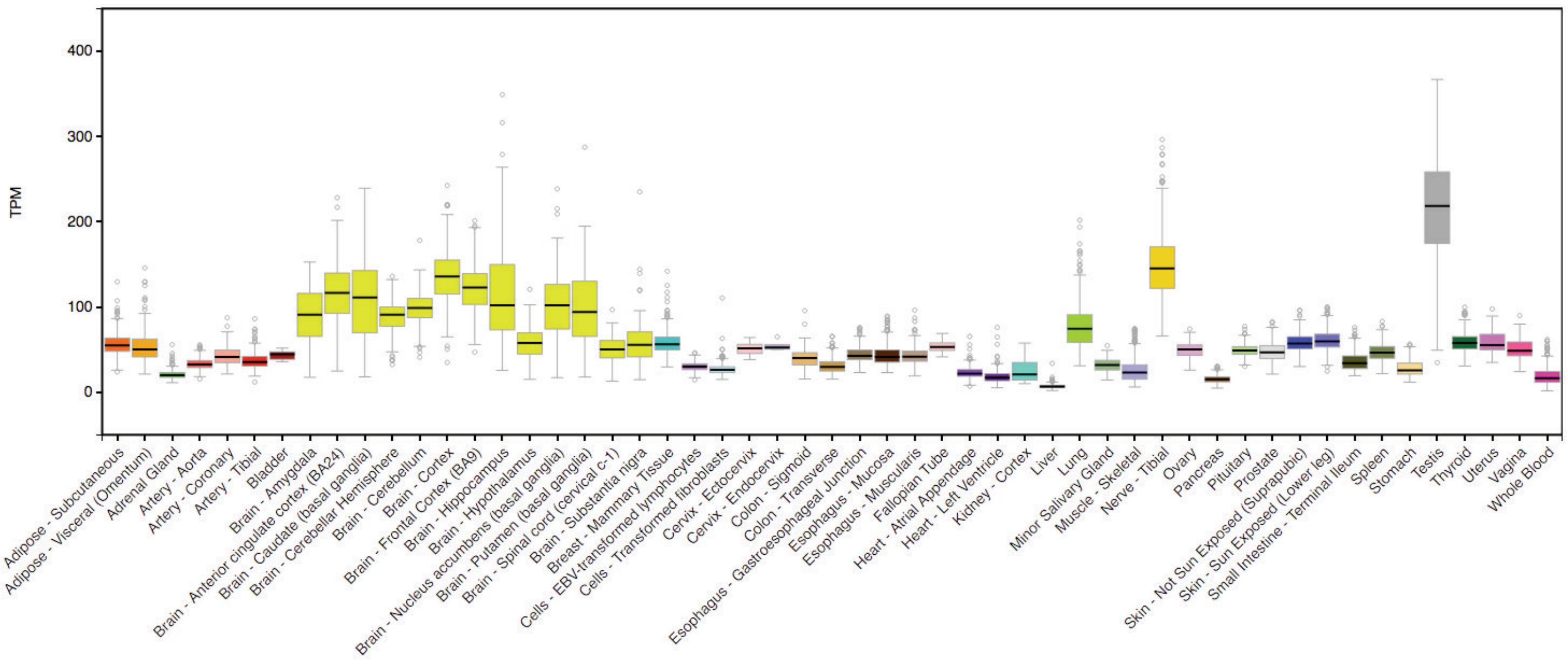

Supplement: Supplementary file 4 — Additional file 4. Case control T-test 524 transcripts. [file 12920_2020_808_MOESM4_ESM.pdf]
